# Current Therapeutic Sequencing in Chronic Lymphocytic Leukemia

**DOI:** 10.3390/hematolrep16020027

**Published:** 2024-04-30

**Authors:** Samir Mouhssine, Nawar Maher, Sreekar Kogila, Claudio Cerchione, Giovanni Martinelli, Gianluca Gaidano

**Affiliations:** 1 Division of Hematology, Department of Translational Medicine, Università del Piemonte Orientale and Azienda Ospedaliero-Universitaria Maggiore della Carità, 28100 Novara, Italy; samir.mouhssine@uniupo.it (S.M.); 20024416@studenti.uniupo.it (N.M.); sreekar.kogila@uniupo.it (S.K.); 2 Hematology Unit, Istituto Romagnolo per lo Studio dei Tumori “Dino Amadori”—IRST IRCCS, 47014 Meldola, Italy; claudio.cerchione@irst.emr.it; 3 Department of Hematology and Sciences Oncology, Institute of Haematology “L. and A. Seràgnoli”, S. Orsola University Hospital, 40138 Bologna, Italy; giovanni.martinelli@irst.emr.it

**Keywords:** chronic lymphocytic leukemia, treatment sequencing, treatment refractoriness, acalabrutinib, zanubrutinib, venetoclax, CAR-T cells

## Abstract

The treatment landscape of chronic lymphocytic leukemia (CLL), the most frequent leukemia in adults, is constantly changing. CLL patients can be divided into three risk categories, based on their IGHV mutational status and the occurrence of *TP53* disruption and/or complex karyotype. For the first-line treatment of low- and intermediate-risk CLL, both the BCL2 inhibitor venetoclax plus obinutuzumab and the second generation BTK inhibitors (BTKi), namely acalabrutinib and zanubrutinib, are valuable and effective options. Conversely, venetoclax-based fixed duration therapies have not shown remarkable results in high-risk CLL patients, while continuous treatment with acalabrutinib and zanubrutinib displayed favorable outcomes, similar to those obtained in *TP53* wild-type patients. The development of acquired resistance to pathway inhibitors is still a clinical challenge, and the optimal treatment sequencing of relapsed/refractory CLL is not completely established. Covalent BTKi-refractory patients should be treated with venetoclax plus rituximab, whereas venetoclax-refractory CLL may be treated with second generation BTKi in the case of early relapse, while venetoclax plus rituximab might be used if late relapse has occurred. On these grounds, here we provide an overview of the current state-of-the-art therapeutic algorithms for treatment-naïve patients, as well as for relapsed/refractory disease.

## 1. Introduction

Chronic lymphocytic leukemia (CLL) is a hematologic neoplasm consisting of the abnormal proliferation and accumulation of mature B cells in lymph nodes, blood, and lymphatic tissues [1]. In Western Europe and in the US, CLL has been reported to be the most common leukemic disease affecting the adult population, with an age-adjusted incidence rate of 4.6–6.3/100,000 person-years [2,3]. The mortality rate of CLL has displayed a decreasing trend during the last few decades, at least in Western countries [3]. In particular, in the US, the CLL mortality rate has decreased from 1.7 to 1.1/100,000 person-years in the period 1992–2020, with a 5-year relative survival gain of 88% between 2013 and 2019 [2]. These data might give rise to the conjecture that CLL may not be a medical challenge anymore, although the disease still represents an unmet clinical need in several contexts. Indeed, CLL is still not curable, and a fraction of patients develop resistance to all available therapies and the disease progresses until death [4]. Moreover, 5–10% of the cases transform to Richter Syndrome, an aggressive lymphoma that usually displays a histologic pattern typical of diffuse large B cell lymphoma (DLBCL) and is associated with refractoriness to standard treatment in the overwhelming majority of cases [5].

Previously, CLL treatment was based on chemoimmunotherapy (CIT) regimens, which combined chemotherapeutic agents with anti-CD20 monoclonal antibodies (mAbs) to obtain a synergistic anti-tumor effect [4]. In the last decade, inhibitors of the B cell receptor (BCR) and B cell lymphoma 2 (BCL2) signaling pathways have become part of the CLL therapeutic landscape, and their use has been steadily increasing thanks to higher efficacy and reduced adverse events compared to CIT [4].

**Figure 1:**
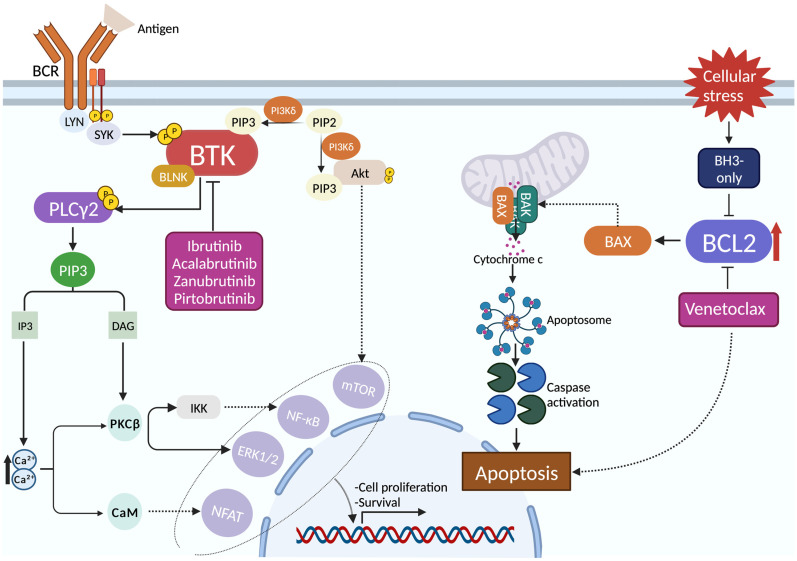
**Molecular activity of BTK- and BCL2- inhibitors in CLL.** The interaction between the B cell receptor (BCR) and its antigen stimulates BTK activity through the SYK protein kinase and the BLNK adapter protein, resulting in the transcriptional activation of genes linked with B cell proliferation and survival. In CLL, the BCR signaling pathway is frequently hyperactivated. Consistently, BTK inhibitors impair this signaling cascade by binding covalently or non-covalently to BTK. On the other hand, the physiological role of the anti-apoptotic protein BCL2 is to sequester BAX, hampering its dimerization with BAK, resulting in heightened cell survival through the impairment of apoptosome formation. Cellular damage causes the inhibition of BCL2 through the BH3-only proteins, stimulating the formation of the BAX-BAK dimer, which promotes cellular death. In the context of CLL, BCL2 is frequently found to be overexpressed, and, for this reason, the BH3 mimetic venetoclax is used to hamper BCL2 activity, resulting in the induction of apoptosis. Abbreviations: SYK, spleen tyrosine kinase; BLNK, B-cell linker; BTK, Bruton tyrosine kinase; PIP3, phosphatidylinositol 3,4,5-trisphosphate; PI3Kδ, phosphoinositide 3-kinase delta; PIP2, phosphatidylinositol 4,5-bisphosphate; PLCγ2, phospholipase C gamma 2; IP3, inositol trisphosphate, DAG, diglyceride; PKCβ, protein kinase C beta; CaM, calmodulin; mTOR, mammalian target of rapamycin; NF-κB, nuclear factor kappa-light-chain-enhancer of activated B cells; ERK, extracellular signal-regulated kinases; NFAT, nuclear factor of activated T-cells; BCL2, B cell lymphoma 2; BH3, BCL2 homology 3; BAX, BCL2-associated X protein; BAK, BCL2 homologous antagonist/killer. Created with BioRender.com (accessed on: 29 October 2023).

The BCR is a surface receptor crucial for antigen recognition and B cell proliferation in the normal physiologic context, while in CLL, the BCR pathway is often constitutively activated (Figure 1) [6]. Blocking the BCR signaling pathway with inhibitors of the downstream kinases Bruton tyrosine kinase (BTK) or phosphoinositide-3-kinase (PI3K) effectively impairs cell survival and proliferation and has been proven to be a game-changing strategy for the management of CLL [4]. More precisely, the most favorable results have been reached with BTK inhibitors (BTKi), which bind covalently (i.e., irreversibly, such as ibrutinib, zanubrutinib, and acalabrutinib) or non-covalently (such as pirtobrutinib) to the BTK molecule (Figure 1) [7]. Consistently, several metanalyses have highlighted the superior efficacy of either the acalabrutinib plus obinutuzumab or ibrutinib plus rituximab/obinutuzumab therapy protocols compared to the other available treatment regimens, especially in comparison to CIT [8,9]. On the other hand, BCL2 is an anti-apoptotic protein that physiologically acts as a positive regulator of cell survival and a negative regulator of cell death (Figure 1) [10]. In CLL cells, the expression of BCL2 is deregulated in the majority of cases, resulting in heightened resistance to apoptosis [4]. The action of BCL2 involves the sequestration of the BAX protein, preventing the formation of the BAX-BAK dimer, which exerts a pivotal role in the apoptosome formation, resulting in impaired apoptosis [10]. Upon binding to BCL2, the BCL2 inhibitor (BCL2i) venetoclax reduces the binding affinity of the anti-apoptotic protein with BAX, thus promoting apoptosis in CLL cells (Figure 1) [11].

Despite the revolutionary role of pathway inhibitors in CLL therapy, treatment refractoriness to these widely employed medicines has been reported, and mechanisms of resistance to both covalent and non-covalent BTKi, as well as to BCL2i, have been described [4]. On these grounds, the aim of this manuscript is to summarize the current treatment sequencing in CLL, providing an overview of the state-of-the-art therapeutic algorithms that can be used for treatment-naïve and relapsed/refractory (R/R) CLL patients.

## 2. Molecular Biomarkers for Directing Treatment Sequencing

The choice of treatment for CLL is highly dependent on three predictive genetic biomarkers, namely the mutational status of the immunoglobulin heavy chain variable (IGHV) genes, the disruption of *TP53*, and, in accordance with certain clinical guidelines, the presence of a complex karyotype (CK) [12].

The mutation status of IGHV genes in CLL stems from the normal physiology and development of B cells in the germinal center. After performing the physiological V(D)J rearrangement that allows the expression of the BCR on the cell surface, B cells in the germinal center undergo somatic hypermutation (SHM), which ensures a high level of diversity across the hypervariable regions of the BCRs of different B cells [13]. CLL can be derived either from a B cell that has experienced the SHM process (in 60% of the cases) or from a B lymphocyte that has undergone maturation in a T-cell-independent manner (in the remaining 40% of the cases) and has not been through SHM [14,15]. The European Research Initiative on CLL (ERIC) suggests a 98% cut-off for IGHV sequence identity to the germline to define the IGHV mutational status [16]. Accordingly, patients with <98% identity are classified as IGHV-mutated, while those with ≥98% identity are considered IGHV-unmutated. IGHV-unmutated patients display a significantly worse outcome if treated with CIT compared to IGHV-mutated cases, while the use of at least some pathway inhibitors, in particular BTKi, can overcome such resistance [4].

The *TP53* onco-suppressor gene is located on the short arm of chromosome 17 (17p) and encodes the p53 protein, which is linked to the cellular response to DNA damage [17]. This so called “guardian of the genome” exerts a pro-apoptotic function consequent to the action of DNA-damaging compounds, including CIT [18]. Consequently, *TP53* disruption by deletion or mutation, or both, increases the resistance of CLL cells to CIT [4]. The frequency of *TP53* abnormalities has been found to increase from ~5% at diagnosis of CLL to 60% at RS development [19]. Furthermore, numerous studies have highlighted inferior outcomes in *TP53*-disrupted CLL patients treated with CIT regimens [4]. To overcome CLL treatment resistance associated with *TP53* disruption, BTKi- and BCL2i-based therapies have been tested in numerous clinical trials. *TP53*-disrupted CLL patients treated with continuous treatment with BTKi displayed favorable outcomes, similar to those obtained in *TP53* wild-type patients [4]. Conversely, fixed-duration therapies with BCL2i have been shown to mitigate, but not to overcome, the disease refractoriness mediated by *TP53* mutation/deletion [20].

Conventionally, CK is defined as the presence of three or more chromosomal aberrations in the same CLL patient [21]. The most common cytogenetic abnormality in CLL is del(13q), which disrupts the *MIR15A* and *MIR16-1* genes, encoding for two miRNA molecules that promote BCL2 proteasomal degradation [11,22]. Consequently, the resulting loss of miR-15a and miR-16-1 causes resistance to apoptosis due to abnormal levels of BCL2 [22]. Other clinically relevant chromosomal abnormalities include del(17p), del(11q), and trisomy 12 [23]. While del(17p) causes *TP53* disruption, del(11q) leads to the loss of *ATM*, a gene encoding the ATM kinase, the key activator of p53 [23,24,25]. Both these genetic lesions translate into the insufficient function of p53, resulting in aberrant cell survival, proliferation and DNA damage resistance [24,25]. Regarding trisomy 12, little is known about the pathophysiological role of this cytogenetic abnormality [26]. CK is associated with resistance to CIT, while contrasting results have been obtained with pathway inhibitors [27]. However, high-CK, defined as ≥5 chromosomal abnormalities, has proven to predict worse outcomes with all available therapies, including venetoclax-based regimens [28].

At diagnosis, after performing fluorescence in situ hybridization (FISH), and DNA sequencing analyses and, if possible, analysis of the patient’s karyotype, CLL patients can be divided into three risk categories, based on their genetic features: (i) low-risk CLL, characterized by IGHV-mutated genes, no del(17p) or *TP53* mutation, and no CK; (ii) intermediate-risk CLL, with IGHV-unmutated genes, no del(17p) or *TP53* mutation, and no CK; (iii) and high-risk CLL, which is characterized by del(17p)/*TP53* mutation and/or CK independent of IGHV mutational status [12]. An integrated prognostic model for the risk stratification of CLL has been proposed and includes a broader array of genetic lesions, namely trisomy 12, del(11q), del(13q), and *TP53*, *BIRC3*, *NOTCH1,* and *SF3B1* mutations [29,30]. Based on the 10-year survival rates, patients might be categorized into four separate prognostic groups: high-risk (marked by *TP53* and/or *BIRC3* abnormalities), intermediate-risk [involving *NOTCH1* and/or *SF3B1* mutations and/or del(11q)], low-risk (characterized by trisomy 12 and wild-type for all genetic anomalies), and very low-risk [indicated solely by del(13q)] [29].

## 3. First-Line Treatment

According to the latest guidelines from the international workshop on CLL (iwCLL), asymptomatic patients should undergo periodic clinical monitoring, while treatment must be reserved for symptomatic and/or progressive disease, summarized as “active disease” [31]. Active disease should match one or more of the following criteria: (i) the occurrence/worsening of anemia/thrombocytopenia; (ii) a massive splenomegaly (≥6 cm below the left costal margin); (iii) a massive lymphadenopathy (≥10 cm in longest diameter); (iv) a lymphocyte doubling time of less than 6 months or a 50% increase in blood lymphocyte count in 2 months; (v) corticosteroid-refractory autoimmune complications; (vi) symptomatic or functional extranodal involvement; and (vii) the occurrence of one of more B symptoms (unwanted weight loss, night sweats, or fever) or severe fatigue [31].

For the first-line treatment of low-risk CLL (IGHV-mutated, no *TP53* disruption and/or CK), the recommended option in Europe is the combination of fixed duration venetoclax plus the anti-CD20 mAb obinutuzumab (VenObi), due to its high efficacy and low toxicity [12,32]. This treatment regimen has been compared to chlorambucil plus obinutuzumab (Chl-O) in low-risk CLL in the CLL14 phase III randomized clinical trial, which enrolled patients affected by comorbidities [32]. VenObi treatment showed a significantly higher efficacy compared to Chl-O, in terms of both MRD reduction (40% vs. 7%, respectively) and 4-year progression-free survival (PFS; 74% vs. 35.4%, respectively) [32]. Similar results have been obtained in young and fit patients in the CLL13 phase III trial [33].

In case of contraindications to obinutuzumab infusion, continuous treatment with a second-generation BTKi, namely acalabrutinib or zanubrutinib, should be evaluated [12]. Acalabrutinib with or without obinutuzumab outperformed the CIT regimen Chl-O in a phase III randomized trial (ELEVATE-TN) that enrolled treatment-naïve CLL patients [34]. Significantly higher 48-month PFS rates (>77%) have been documented for acalabrutinib-containing arms compared to the Chl-O arm (~25%). Remarkably, this superiority in terms of efficacy was maintained in IGHV-unmutated patients (intermediate-risk CLL) and *TP53*-disrupted patients (high-risk CLL) [34]. In a phase III randomized study (SEQUOIA), similar results were obtained with zanubrutinib monotherapy in low-risk CLL patients, which displayed significantly higher efficacy compared to bendamustine plus rituximab (BR) [35]. Although the first-generation BTKi ibrutinib, with or without obinutuzumab, is burdened by a higher cardiotoxicity profile with no efficacy advantage, it might be used in cases of the unavailability of second-generation BTKi [12,36]. Recently, the time-limited combination of orally administered ibrutinib and venetoclax (I + V) has been approved by the European Medicines Agency (EMA), making this regimen a viable option for first-line CLL therapy [12]. The safety and efficacy of I + V have been assessed in two randomized phase II trials (GLOW and CAPTIVATE studies), where I + V-treated patients achieved higher PFS rates compared to CIT-treated patients not only in low-risk CLL, but also in intermediate- and high-risk disease [37,38]. In geographic contexts in which therapy with pathway inhibitors is not accessible or affordable, a CIT regimen, such as Chl-O, BR, or FCR (fludarabine, cyclophosphamide and rituximab), should be used [12,39].

The first-line treatment of intermediate-risk CLL (IGHV-unmutated, no *TP53* disruption and/or CK) relies on the use of acalabrutinib with or without obinutuzumab or zanubrutinib or, if these are unavailable, ibrutinib with or without obinutuzumab [12]. The rationale for this therapeutic approach is based on several phase III randomized trials, where no difference in clinical outcomes was observed between IGHV-mutated and IGHV-unmutated CLL [34,35,40]. In cases of severe cardiac comorbidities, VenObi is recommended regardless of IGHV status [12]. As a third option, I + V can be used based on the relatively favorable results obtained in IGHV-unmutated patients, as mentioned above [12,37,38].

For high-risk CLL patients (*TP53* disruption and/or CK), first-line treatment is based on continuous BTKi therapy, preferably with acalabrutinib or zanubrutinib [12]. Acalabrutinib, with or without obinutuzumab, outperformed Chl-O in *TP53*-disrupted CLL in the ELEVATE-TN trial, with a 48-month PFS of 76.2% [34]. In the SEQUOIA trial, high-risk CLL patients treated with zanubrutinib achieved a 24-month PFS of 87%, similar to that obtained in low-risk patients (85%) [35]. If the patient is unsuitable for BTKi, fixed duration VenObi might be an option, although suboptimal results with this regimen were obtained in high-risk CLL in the CLL14 trial [32]. Time-limited I + V therapy may represent a valid alternative, based on the promising efficacy data from the CAPTIVATE study [12,38]. Nonetheless, I + V is still under evaluation for the first-line treatment of high-risk CLL, and further investigations with larger study cohorts are needed [38]. In exceptional cases, the combination of the PI3K inhibitor (PI3Ki) idelalisib and rituximab can also be considered, taking into account the high risk of severe pneumonia and immune-mediated side effects after treatment administration [12].

## 4. Treatment of Relapsed/Refractory Disease

The treatment of R/R CLL depends significantly on the previously administered therapy [12]. If disease progression occurs after CIT, fixed duration venetoclax plus rituximab (VenR) regimen or continuous treatment with a covalent BTKi are recommended [12]. In the MURANO study, a randomized phase III clinical trial, VenR was superior to BR in R/R CLL, showing higher PFS and overall survival (OS), even in *TP53*-disrupted or CK patients [41]. Among BTKi, acalabrutinib and zanubrutinib achieved the best clinical outcomes in terms of PFS and OS in phase III trials, outperforming ibrutinib and CIT in CLL treatment, regardless of the risk category [42].

First-line treatment with pathway inhibitors, in particular with BTKi and BCL2i, may lead to acquired resistance to these therapeutic agents [4]. Although data on the optimal treatment sequencing in CLL refractory to pathway inhibitors are still insufficient, several mechanisms of treatment refractoriness have been identified [4,12]. In detail, the most frequent mechanism of acquired resistance to covalent BTKi is the occurrence of point mutations of the cysteine residues 481 of BTK, such as C481S and C481R, which lead to the impairment of covalent binding of the drug to its target [4]. Other mechanisms of resistance are independent from BTK mutational status and include point mutations of the downstream signaling molecule PLCγ2, del(8p) and genetic lesions of *BIRC3* and *NFKBIE* [4,43]. Consistently, in covalent BTKi-resistant CLL patients, the recommended treatment option is the combination of fixed-duration VenR, which showed favorable outcomes even in *TP53*-disrupted patients [12,41,44]. An innovative but not yet approved therapeutic approach makes use of non-covalent BTKi, such as pirtobrutinib, which is capable of overcoming point mutations affecting C481 [4,45]. However, refractoriness to non-covalent BTKi is still possible via BTK-independent mechanisms [4,46]. Moreover, a few point mutations in the tyrosine kinase domain of BTK (e.g., T474I and L528W) lead to non-covalent BTKi resistance. In particular, the L528W mutation can also arise after covalent BTKi treatment, causing cross-resistance between covalent and non-covalent inhibitors [4]. Consequently, in the near future, DNA sequencing may be a useful tool to test CLL patients who relapsed after covalent BTKi, in order to make effective treatment decisions.

Up to 50% of acquired resistance to BCL2i after venetoclax treatment in CLL is due to the substitution of glycine 101 to valine (G101V) within the amino acid sequence of BCL2 [47]. This mutation alters the target binding domain of venetoclax, reducing the cellular response to the drug [4]. Recently, several additional genetic lesions have been detected in R/R CLL patients treated with venetoclax, such as del(8p), 1q gain, and BCL2 amino acid substitution D103Y [47,48]. In the case of an early relapse after venetoclax-based regimens, therapy is based on continuous treatment with a covalent BTKi [12]. Conversely, if a late relapse occurs, a valuable option may be time-limited VenR, which was found to be effective after venetoclax-containing treatment regimens in late-relapsed CLL patients [12,49,50].

Furthermore, although burdened by high toxicity, an idelalisib plus rituximab treatment regimen may be exceptionally useful following CLL refractory to both BTKi and BCL2i [12,51]. Due to its high mortality, allogeneic hematopoietic stem cell transplantation might be considered as a last option in patients refractory to all recommended therapies [4,12].

The National Comprehensive Cancer Network (NCCN) Clinical Practice Guidelines in Oncology (NCCN Guidelines^®^) continue to list treatments that are not commonly employed for the management of R/R CLL following second-generation BTKi and BCL2i failure, primarily due to their reduced effectiveness and/or increased toxicity [30]. These therapies encompass the use of the anti-CD52 mAb alemtuzumab with or without rituximab, the PI3K inhibitor duvelisib, the immunomodulatory drug lenalidomide with or without rituximab, and high-dose methylprednisolone in combination with an anti-CD20 mAb [30,52,53,54,55,56,57,58,59,60,61]. Although still recommended by NCCN Guidelines^®^, in Europe these options are considered as the last line of therapy, with a preference towards enrolling patients in clinical trials investigating third-generation BTKi or other emerging treatment strategies [12,30].

## 5. Novel Strategies for the Treatment of R/R CLL

Several novel anti-tumor medicines are under development in order to overcome treatment resistance to BTK and BCL2 inhibitors, including immunotherapeutic agents and proteolysis-targeting chimeras (PROTACs) [4]. Immunotherapy represents a therapeutic approach aimed at modulating the immune system to elicit an immune-mediated response against neoplastic cells [62]. CLL, due to its peculiar immune microenvironment and promotion of immune dysfunction, initially appeared as a promising candidate for immunotherapy [63]; however, this approach has encountered challenges for the same reasons [64]. Numerous innovative therapeutic agents are currently the subjects of investigation in the context of CLL immunotherapy. Specifically, these agents include chimeric antigen receptor (CAR)-T/natural killer (NK) cells, as well as immune checkpoint inhibitors [64].

CAR-T cells are characterized by the expression of surface receptors, CARs, consisting of a fusion of an extracellular domain responsible for antigen binding, a signaling CD3ζ domain, and one or more intracellular co-stimulatory subunits, such as CD28 or 4-1BB [65,66,67]. CAR-T treatments assessed in the context of CLL involve the use of cellular products designed to specifically target the surface antigen CD19 [68]. Although anti-CD19 products have achieved a relatively promising overall response rate (ORR) of up to 100% in CLL treatment, the average rate of complete remission (CR) is approximately 30% [69]. These results appear to be even lower when compared to the outcomes observed in acute lymphoblastic leukemia (ALL) and DLBCL [70,71,72].

A possible mechanism of resistance could be the selection of CLL clones with reduced expression of CD19 under the pressure of anti-CD19 CAR-T cell therapy [73]. Consequently, a potentially effective strategy to overcome resistance to anti-CD19 CAR-T cells might involve the development of cellular therapies which target B cell surface molecules that are different to CD19 [74]. On these grounds, an innovative CAR-T construct (MC10029) has been designed to target the B cell-activating factor receptor (BAFF-R), a surface receptor that promotes B cell proliferation and maturation through the activation of the NF-κB pathway [74,75].

Another mechanism of resistance to CAR-T cell treatment is represented by the dysregulation of the PD-1/PD-L1 axis, which is composed of PD-1, a surface molecule expressed by normal T cells, and its ligand PD-L1, typically situated on the surface of antigen-presenting cells [4,76]. This interaction sets off intracellular signaling pathways via the PD-1 domain, ultimately culminating in the inhibition of the PI3K/Akt and MAPK pathways, resulting in T cell exhaustion [77,78]. In CLL, PD-1 has been found to be overexpressed on T cells, while PD-L1 is overexpressed on the outer membrane of neoplastic B cells, generating an immunotolerant environment that enables the tumor to elude apoptosis [79]. Monotherapy with pembrolizumab, an anti-PD-1 mAb, displayed a lack of efficacy in relapsed CLL patients, with an ORR of 0% [80]. Since increased IL-10 serum levels have been found to play a role in promoting immune checkpoint inhibitor resistance, a combination approach involving immune checkpoint inhibitors along with IL-10 inhibition has been explored [4]. Recently, promising initial results have been achieved for simultaneous treatment with IL-10 suppression agents and pembrolizumab in murine xenograft models of CLL [81].

Additionally, as resistance to immunotherapy in CLL is predominantly attributed to T cell exhaustion, immunotherapeutic strategies employing CAR-NK cells have recently been explored for CLL treatment [69,82]. CAR-NK cells are genetically modified NK cells derived from healthy donors, sourced from umbilical cord blood or peripheral blood [83]. These cells are engineered to express a CAR on their surface, harnessing the NK cytotoxic response to target tumor cells [83]. While early encouraging outcomes have surfaced in ongoing phase I/II trials (NCT04245722, NCT03056339, NCT04796675) employing CAR-NK cells in CLL treatment, further assessment is imperative to comprehensively evaluate the safety and efficacy profiles of this innovative therapeutic paradigm [69].

The challenge of BTK inhibitor resistance could potentially be met through the use of proteolysis-targeting chimeras (PROTACs) [4]. PROTACs, representing a novel category of small molecules, function by concurrently recruiting the target protein and an E3 ubiquitin ligase, triggering the ubiquitination process that ultimately leads to the proteasomal degradation of the target protein [84]. PROTACs, such as NX-2127, have demonstrated their efficacy both in vitro and in vivo by effectively causing the degradation of BTK in CLL cells with an amino acid substitution at C481 of BTK [85,86]. Recently, the clinical outcomes of an ongoing phase I trial (NCT04830137) involving NX-2127 in the first-in-human setting have been disclosed [86]. All participants of the study had previously undergone treatment with covalent BTK inhibitors and/or venetoclax. Notably, NX-2127 led to a remarkable ORR of 33% in the evaluable patients, as assessed at a median follow-up duration of 5.6 months [86]. These findings provide robust support for the utilization of BTK degraders in patients resistant to multiple lines of therapy, irrespective of their BTK or BCL2 mutation status.

Although still in a preliminary stage, ongoing clinical trials are investigating novel therapeutic agents for the treatment of R/R CLL, including the CD20xCD3 bispecific antibody (bsAb) epcoritamab (NCT05791409, NCT04623541, NCT03625037, NCT04542824), the ROR1xCD3 bsAb NVG-111 (NCT04763083), the anti-ROR1 mAb cirmtuzumab (NCT03088878), the drug-immunoconjugate zilovertamab vedotin (NCT05458297), and the radioconjugate phospholipid ether iopophosine I-131 (NCT02952508).

## 6. Conclusions and Perspectives

In the last few years, the treatment landscape of CLL has been revolutionized by the advent of pathway inhibitors, in particular BTKi and BCL2i. For the first-line treatment of CLL, both VenObi and second generation BTKi can be adopted on the basis of a patient’s risk category and comorbidities. The development of acquired resistance to pathway inhibitors represents an ongoing clinical challenge, and the optimal treatment sequencing of R/R CLL relies on the use of acalabrutinib/zanubrutinib or VenR, based on prior therapy and duration of response to the previous treatment. Although non-covalent BTKi have demonstrated high efficacy in CLL refractory to covalent BTKi, several mechanisms of resistance to these novel agents have been described. For this reason, a precision medicine approach may be potentially warranted, using DNA sequencing to display the mutational profile of a given single patient in order to make effective therapeutic decisions.

Finally, novel treatment strategies to overcome refractoriness to pathway inhibitors are currently under evaluation, including BTK degraders and immunotherapy with CAR-T or CAR-NK cells, but data on the effectiveness of these therapeutic approaches are still insufficient.
